# The impact of cigarette prices on smoking participation and tobacco expenditure in Vietnam

**DOI:** 10.1371/journal.pone.0260415

**Published:** 2021-12-14

**Authors:** Cuong Viet Nguyen, Thu Thi Le, Nguyen Hanh Nguyen

**Affiliations:** 1 International School, Vietnam National University, Hanoi, Vietnam; 2 IPAG Business School, Paris, France; 3 HealthBridge Foundation of Canada, Hanoi, Vietnam; Keck School of Medicine of the University of Southern California, UNITED STATES

## Abstract

Vietnam is one of countries with the highest number of smokers in the world and the high smoking prevalence among men in the region. Although the real cigarette prices increased by around 4% during the 2010–2015 period, the prevalence of daily cigarette smoking among men decreased slightly from 31.3% to 30.7% during this period. This raises the question of whether cigarette consumption is sensitive to price. In this study, we estimated the effect of cigarette prices on smoking participation and tobacco expenditure in Vietnam. We found that a one-percent increase in the real cigarette price reduced the probability of cigarette smoking among males by 0.08 percentage points (95% CI from -0.06 to -0.10), equivalent to the price elasticity of the smoking prevalence at -0.26 (95% CI from -0.16% to -0.33%). Using this estimate, we predict that if the cigarette price is increased by 10%, the daily cigarette smoking prevalence among men would decrease from 30.7% to 29.9% and the number of male smokers would decline by around 270 thousand. Higher cigarette prices also reduced per capita tobacco expenditure of households. A one-percent increase in the cigarette price decreased per capita expenditure on tobacco consumption expenditure of households by 0.43 percent (the 95% CI from -0.029 to 0.822). This finding suggests that raising tobacco taxes and prices can be an effective measure to reduce tobacco use.

## Introduction

The adverse effects of tobacco consumption on health have been well documented (e.g., see recent review from [1, 2]). Yet, there are still around 1.1 billion people who are using tobacco products [3]. An effective policy to reduce smoking is to increase the price of tobacco products through tax (e.g., see [4–6]). Existing reviews demonstrate that increasing the prices of tobacco products reduces smoking prevalence and tobacco consumption [7, 8]. Although there is consensus on the negative effects that tobacco price has on the prevalence of smoking, the magnitude of the effects varies across empirical studies [8, 9]. For example, a review by Guindon and colleagues [9] shows that the own-price elasticity for cigarettes varies from -0.1 to -1.4 in Latin America and the Caribbean countries. Main reasons for this variation include differences in country contexts, estimation methods, and data [9]. The heterogeneity of the findings call for more empirical research on the effects of tobacco price on tobacco consumption.

Vietnam has very high smoking rates among men; in 2015, the daily smoking rate among men was 38.7% (estimated from the 2015 Global Adult Tobacco Survey). Moreover, there are a large proportion of people exposed to second-hand smoke. In 2016, 63% of the population reported living in households where tobacco is consumed (estimated from the 2016 Vietnam Household Living Standard Survey). The real cigarette price increased by about 4% between 2010 and 2015. However, the prevalence of daily tobacco smoking among men was unchanged during this period, at 38.7%, while the prevalence of daily cigarette smoking among men declined marginally from 31.3 percent to 30.7 percent (these figures are estimated using data from Global Adult Tobacco Surveys 2010 and 2015). This raises the question as to whether tobacco consumption is sensitive to tobacco price. Information on price elasticity of tobacco expenditure is particularly important for policy makers to predict to what extent an increase in tobacco price, induced by tobacco tax, can reduce tobacco consumption. In this study, we estimate the effect of cigarette price on cigarette smoking participation among males and tobacco consumption expenditure of households in Vietnam, using recent data from Global Adult Tobacco Surveys (GATS) in 2010 and 2015, and Vietnam Household Living Standard Surveys (VHLSS) from 2010 to 2016. To address the endogeneity bias of the cigarette price, we use province fixed-effects and an instrumental approach, in which the instrument for the current cigarette price is lags of cigarette price.

Our study is expected to contribute to the greater body of evidence on this topic by providing empirical evidence on the effect of cigarette price on smoking participation of individuals in Vietnam, in addition to household tobacco expenditure. Vietnam is an interesting setting to study this phenomenon as it has the 3^rd^ highest smoking rate in Southeast Asia, after Indonesia and Laos [10]. Additionally, it is estimated that nearly one-third of Vietnamese citizens are exposed to second-hand smoke [11]. Tobacco consumption is estimated to cause 40,000 deaths each year in Vietnam [12]. Annual economic losses due to tobacco consumption are estimated to be nearly 1% of the national GDP [13]. Economic losses include the cost of treatment for five tobacco-related diseases, employment losses due to illnesses, and premature deaths caused by smoking. Thus, understanding to what extent the tobacco price can reduce tobacco consumption is important for designing tobacco taxation policies. Findings from this study may be relevant for other Asian countries with similar context to Vietnam, such as Indonesia and Laos.

There are several studies on the effect of cigarette price on smoking behaviors in Vietnam. Using the Vietnam Living Standard Survey 1998, Eozenou and Fishburn estimates the price elasticity for cigarette demand in Vietnam to be around -0.53 [14]. Also using the same data set, Laxminarayan and Deolalikar [15] found that higher pricing of cigarettes significantly and negatively associated with the initiation of cigarette smoking. Guindon [16] found that higher pricing of tobacco delays the onset of smoking among young people. Recently, Nguyen and colleagues [17] also found cigarette price to have a negative effect on the probability of the smoking initiation. Compared with previous studies in Vietnam, our study looks at smoking participation among men in addition to tobacco consumption of households using recent data sets. We also examine the heterogeneous impacts of cigarette prices on smoking and tobacco expenditure among different population sub-groups including: urban and rural people, Kinh and ethnic minorities, people of different age groups, people with varying education and wealth levels.

The paper is structured as follows. The second and third section present data sets and descriptive analysis of smoking in Vietnam, respectively. The fourth section presents the estimation strategy. The fifth section discusses the empirical findings. Finally, the sixth section concludes.

## Data sources

In this study, we used three sources of data. The first data source, which is used to measure the smoking participation of men, is from the 2010 and 2015 Global Adult Tobacco Surveys (GATSs) of Vietnam. GATSs were conducted by General Statistics Office of Vietnam (GSO) with technical support and funding from the World Health Organization in 2010 and 2015. They are nationally representative surveys of men and women aged 15 years and older in all 63 provinces of Vietnam. The number of individuals who completed the questionnaires are 9,925 and 8,996 in 2010 and 2015, respectively (the overall response rate was 92.7% in 2010 and 95.8% in 2015). The GATSs Vietnam questionnaire consists of eight sections, including background characteristics, tobacco use, smoking cessation, second-hand smoke, economics, media, and knowledge, attitude, and perceptions.

The second data source is the Vietnam Household Living Standards Surveys (VHLSSs). The VHLSSs are conducted every two years by the GSO since 2002. In this study, we use the VHLSSs from 2006 to 2016. We do not use the 2002 and 2004 VHLSSs, since data on cigarettes are not available for this period. VHLSSs are statistically representative of national, urban and rural, and regional levels. The number of households in each VHLSSs before 2010 is approximately 9,200, while the number of households in each VHLSSs from 2010 is approximately 9,400.

The VHLSSs collect detailed information on individuals and households. Individual-level information is collected regarding health, education, employment and demographic characteristics of the population. Household-level information that the survey collects includes income, consumption expenditure, housing conditions and durable ownership. Additionally, the VHLSSs collect information from households regarding their expenditure on tobacco during the past 12 months. However, the VHLSSs do not collect data on individual expenditure of tobacco, neither the type of tobacco products consumed, such as cigarettes or water pipes. The VHLLSs ask households to report their total consumption expenditure of purchased and homemade tobacco.

The third data source is the cigarette price. In this study, we measure the cigarette price by the detail price of Vinataba cigarette (brand-named Vinataba per pack of 20’s cigarette). Pricing data are available at the provincial level from 2001 to 2016. Vinataba is an abbreviation of the Vietnam National Tobacco Corporation, which is the largest tobacco company in Vietnam, with a cigarette market share of 55% in 2015 [18]. Vinataba cigarettes are a popular brand, under medium price segment and accounts for 9.1% of cigarette volumes in 2015 [18]. S1 Fig presents the nominal and real prices of Vinataba cigarettes (thousand VND per pack) and the rate of daily tobacco smoking over time. From 2001 to 2015, the nominal Vinataba cigarette price increased by 138%, from 8,738 VND/pack to 20,815 VND/pack. Because of relatively high inflation rates in Vietnam, the real price (after adjusted to the 2001 price using overall CPI) increased at a much smaller rate, by approximately 20% during the same period of time.

In Vietnam, GSO constructs the tobacco CPI from the prices of three main tobacco products including Vinataba cigarette, 555 cigarette, and water-pipe tobacco, and a cheap cigarette brand (such as Thang Long or Bastor brand), which is the most popular in each province. The data on the tobacco CPI are only available for 30 provinces, while data on the price of Vinataba cigarette are available for all the 63 provinces of Vietnam. In this study, we use the price of Vinataba cigarette to increase the sample size and avoid sample selection bias. Moreover, using the cigarette price (instead of CPI) we can compute the price elasticity of tobacco demand. S2 Fig shows a strong correlation between the tobacco CPI and the Vinataba cigarette prices with a correlation coefficient of 0.78. We expect that the Vinataba cigarette prices can be representative for the tobacco CPI.

## Smoking and tobacco expenditure in Vietnam

Although more and more information on the harmful effects of smoking has been reported, the proportion of smokers has not decreased in Vietnam. The prevalence of smoking any products among people aged 15 and over was 23.8% in 2010 and 22.5% in 2015. Vietnam has some of the highest smoking rates in Asia. Compared with other Southeast Asian countries, Vietnam has the 3^rd^ highest tobacco smoking rates after Indonesia and Laos [10]. Like many other Asian countries, smoking in Vietnam is most common among men. The tobacco smoking prevalence of men was 45.3% in 2015, and was only 1.1% among women in 2015. The proportion of daily smokers in Vietnam is slightly lower. Daily smokers are those who smoke any tobacco products every day (defined based on the questionnaires of GATSs). In 2015, 38.7% of men were daily tobacco smokers, and 30.7% of men were daily cigarette smokers. Manufactured cigarettes and water pipes are the two main tobacco products consumed in Vietnam. Among male smokers, 81% smoked cigarettes and 30% smoked traditional water pipes in 2015.

**Fig 1:**
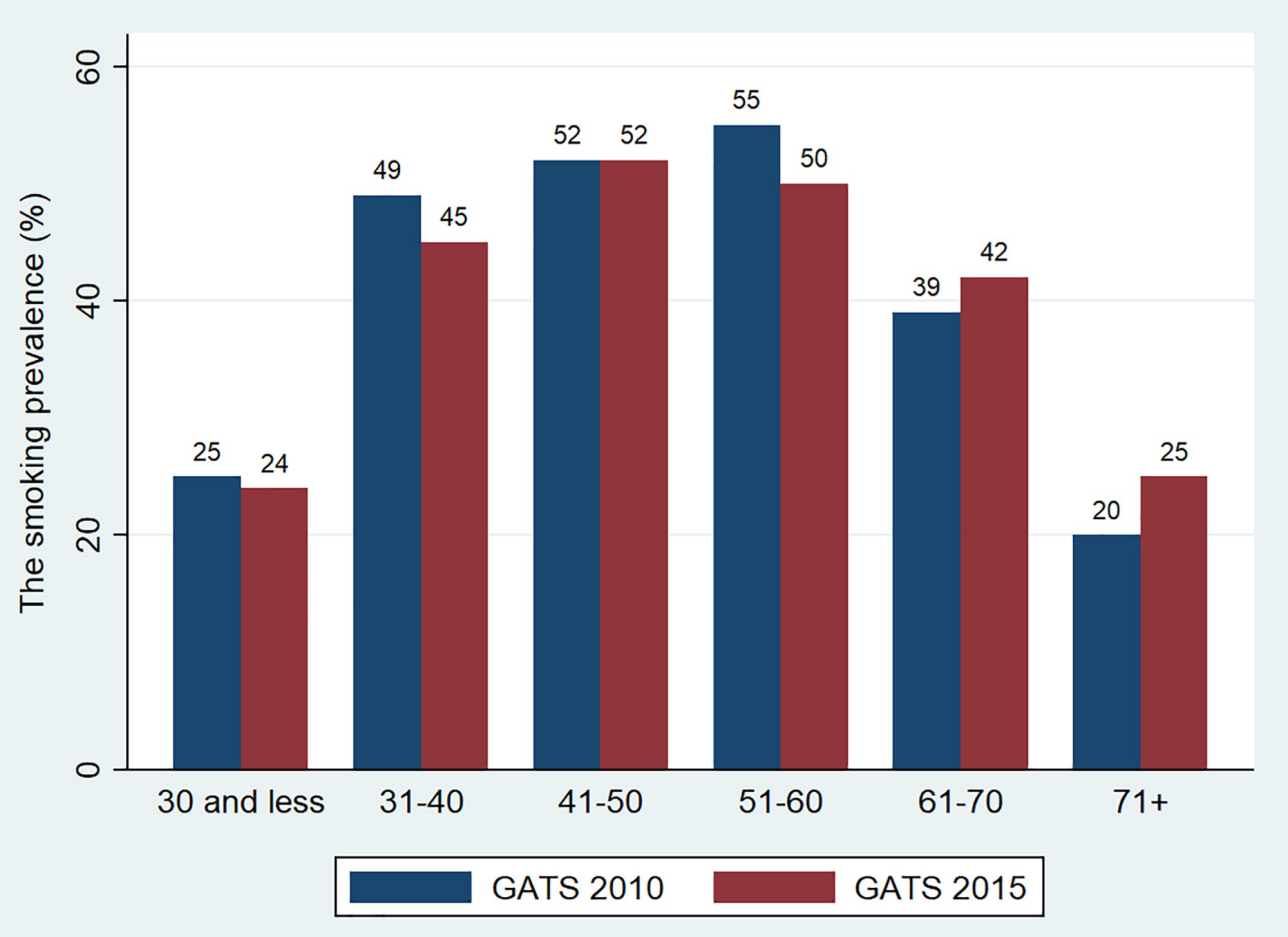
The prevalence of daily tobacco smoking of males by age groups. Note: The figure uses the sample of male individuals aged from 15. Source: Estimation from GATS 2010 and 2015.

Fig 1 presents the daily tobacco smoking rate of men by age group in 2015. In this study, we focus on smoking among men, since the smoking prevalence among women is very low. Smoking prevalence are highest among middle-aged men, with 53% for men aged 25–44 and 55% for men aged 45–64. Middle-aged men have higher incomes and are more likely to afford smoking than younger and older people. Smoking prevalence are lower among younger people (15–24 years), at approximately 24.3%. Smoking prevalence remains rather high among older people. Around one-third of men aged 64 and over smoked in Vietnam. S1 Table presents the prevalence of tobacco smoking and cigarette smoking by different characteristics among men.

**Fig 2:**
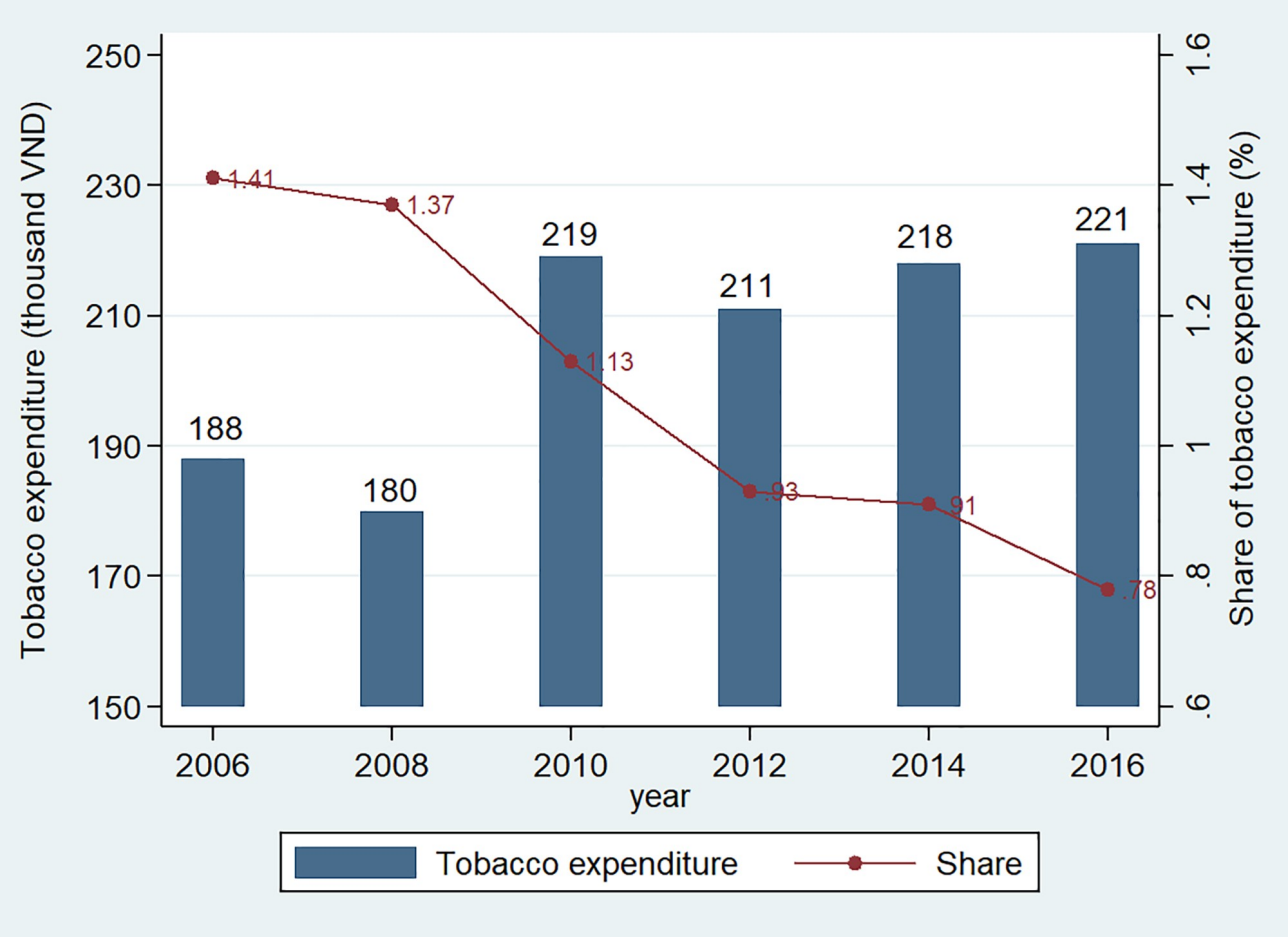
Tobacco consumption expenditure of households. Note: The tobacco expenditure is measured in the 2016 price. Source: Authors’ estimation from VHLSSs.

Using VHLSSs, we estimate the per capita tobacco consumption expenditure during the past 12 months (Fig 2). We adjust consumption expenditure to the 2016-year price using overall Consumer Price Index (CPI). Tobacco expenditure was quite stable between the years of 2010 and 2016. However, the share of tobacco expenditure in the total expenditure of households decreased over time. It decreased from 1.41% in 2006 to 1.13% in 2010 and to 0.78% in 2016. S2 Table presents the per capita tobacco expenditure and its share in the total household expenditure of different population sub-groups.

It should be noted that there is a large gap in per capita tobacco expenditure between 2008 and 2010, since there was a change in the VHLSSs questionnaires before 2010 and those since 2010. In the VHLSSs before 2010, households were asked for total tobacco expenditure in the past 12 months, while in the VHLSSs since 2010 households were asked for tobacco expenditure from the last 30 days, which is then multiplied by 12 to estimate annual tobacco expenditure.

## Method

### Ethics

This study uses secondary data from Global Adult Tobacco Surveys (GATS) in 2010 and 2015, and Vietnam Household Living Standard Surveys (VHLSS) from 2010 to 2016. These data were provided by General Statistics Office of Vietnam and the World Health Organization. The data are fully anonymized, and there are no ethical issues and conflicts of interest related to the respondents in the surveys.

### Estimation strategy

The first objective of this study is to measure the effect of tobacco price on daily cigarette smoking participation among male adults (aged from 15). We used the following regression model:

$$Y_{i,j,t}=\beta_{0}+Log\left({\text{Price}}_{j,t}\right)\beta_{1}+X_{i,j,t}\beta_{2}+P_{j}\beta_{3}+T_{t}\beta_{4}+\varepsilon_{i,j,t},$$ (1)

where *Y*_*i*,*j*,*t*_ is the status of daily smoking of male individual *i* in province *j* in year *t*. It is equal to 1 for daily smokers and 0 otherwise. *Log*(Price_*j*,*t*_) is logarithm of the average price of Vinataba cigarette pack of province *j* in year *t*. This price is adjusted to the 2001 price using overall provincial-level CPI. *X*_*i*,*j*,*t*_ is a set of individual-level control variables, and *P*_*j*_ is a vector of province-level control variables. *T*_*t*_ is a vector of the time dummy variables.

The individual-level control variables include age, education, Kinh majority, urban dummy and wealth index. There are no data on income or expenditure of individuals in this model, and we construct a wealth index to measure the living standard of individuals. Higher value of the asset index means a higher access to the assets. The asset index is standardized to have a mean of zero and a standard deviation of one. We follow the principal components approach of Filmer and Pritchett [19] and Kolenikov et al. [20] to compute a wealth index. According to this approach, an index is constructed as the first principal component of a vector of assets of households such as durables and housing conditions. The principal component approach defines a wealth index in terms of the first principal component of the variables used. The wealth index, denoted by *A*_*j*_, for household *j* is computed as follows:

$$A_{j}=\sum_{p}a_{p}(\frac{x_{pj}-{\bar{x}}_{p}}{S_{p}})$$

where *x*_*p*_ denotes the asset *p*, and $\bar{x}$ denotes a mean of households in the sample. *s* is a standard deviation of asset *x*_*p*_, and the *p*-dimensional vector of weight *a* is chosen to maximize the sample variance of *A*, subject to $\sum_{p}a_{p}^{2}=1$. The weight *a* is also called the vector of scores of asset variables, which can be estimated using principal component analysis. Based on the availability of data. The assets used to compute the wealth index consists of including flush latrine, telephone, television, radio, fridge, car, washing, air-conditioning, motorboat, electricity generator, grinder, computer, and internet connection. We also control for the year, and province dummies. The province-level control variables including overall CPI, per capita income, and log of population of density. S3 Table presents the definition of the control variables.

Model (1) is estimated using data from GATSs 2010 and 2015. We used data representing the smoking history of individuals and to compute the smoking status of individuals in a given year, using the GATS surveys. We constructed cohort data on smoking status for all individuals in the datasets. For example, for *n* male observation in the 2015 GATS, we know their smoking status not only in 2015 but also in previous years by combining information on their age to start and stop smoking. Suppose a man started smoking in 2012 but stopped smoking since 2014, then his smoking status is ‘not smoking’ before 2012, then ‘smoking’ in 2012 and 2014, and ‘not smoking’ in 2014 and 2015. By expanding observations over time, we have panel data on the smoking status, demographic variables such as age and gender, and the provincial-level cigarette prices. Using the cohort data increases the number of observations over time, which allowed us to have more variation in the cigarette price over time and across provinces. Since data on cigarette prices are available from 2001 to 2016 and the latest GATS is from 2015, we limited data on smoking among individuals to 2001 to 2015.

Since individuals are expanded over years, we need to cluster the standard errors at the individual level over time. We adopt the multiway clustering technique of Cameron and colleagues [21], which allows us to address the correlation of errors within individuals across yeas and between individuals within enumeration areas simultaneously. In the Stata, we use ivreg2 command which allows for regressions with two-way level cluster.

The second objective of this study is to examine the impact of the cigarette price on the tobacco expenditure of households. Using the household-level data from VHLSSs, we estimated the following model:

$$Log(C_{k,j,t})=\theta_{0}+Log\left({\text{Price}}_{j,t}\right)\theta_{1}+{\text{X}}_{k,j,t}\theta_{2}+P_{j}\theta_{3}+T_{t}\theta_{4}+u_{k,j,t},$$ (2)

where *Log*(*C*_*k*,*j*,*t*_) is logarithm of per capita expenditure on tobacco products during the past 12 months of household *k* in province *j* in year *t*. *Log*(Price_*j*,*t*_) is logarithm of the average price of Vinataba cigarette pack of province *j* in year *t*. *X*_*k*,*j*,*t*_ is a set of household-level control variables, and *P*_*j*_ is a vector of province-level control variables. *T*_*t*_ is a vector of the time dummy variables. The price elasticity of tobacco expenditure is measured by *θ*_1_. Eq (2) is widely used to model tobacco demand and the price elasticity of tobacco demand (e.g., see [22]).

Explanatory variables include household composition, characteristics of household head, log of per capita income, and geographic variables. We tend to use exogenous explanatory variables, since the explanatory variables should not be affected by the treatment variable [23, 24]. The summary statistics of the outcome and control variables are presented in S4 Table.

An important issue in estimating the effect of the tobacco price is endogeneity bias. The cigarette price is jointly determined by demand and supply of cigarettes. A higher cigarette price reduces demand for cigarettes, but a lower cigarette demand will subsequently lead to a lower cigarette price. In addition, the cigarette price can be also correlated with unobserved variables, especially province-level variables. To address this issue, we controlled for province dummies in the regression (province fixed effects). The cigarette price is at the provincial level, and controlling for province fixed-effects is expected to address the endogeneity bias. In addition, we used instrumental variable regression, in which the instrument for the price of cigarette is the lagged cigarette price. The instrumental variable regression is a traditional econometric method that can provide a consistent estimate of the effect of an endogenous variable (the price of cigarette in this study). An instrument is required to be correlated with the endogenous variable but not the error term in the outcome equation (for detailed presentation of this method, see any econometric textbook, e.g., [25]).

A widely-used instrument for the cigarette price is lags of cigarette price (e.g. [22, 26]). In this study, we use cigarette price lagged by one, two and three years as the instrumental variables for the current cigarette price. The main advantage of using lags as the instrumental variables is that they are strongly correlated with the current price, therefore avoiding the weak instrument bias problem. However, it is possible that the instruments can be correlated with the error term in the outcome equation. Using different lags of cigarette price, we can conduct an over-identification test of the validity of the instruments [27, 28]. A better instrumental variable for cigarette price is cigarette taxes. For the case of Vietnam, the special consumption tax on cigarettes increased only two times (in 2006 and 2008) during the 2006–2016 period. Moreover, there is a national cigarette tax rate in Vietnam. There are no variations in the tax rate across provinces. Thus we cannot measure the effect of the cigarette tax rate on cigarette price and use it as the instrumental variable for the cigarette price. Another possible instrumental variable for cigarette price is the lead price. In this study, we tried this instrumental variable, but the estimation result shows a positive and insignificant effect of the cigarette price. It is possible that the lead price can be affected by the cigarette demand in the current period. If people consume less (or more) cigarette in the current period, the price of cigarettes can be lower or higher in the next period. The exclusion assumption can be violated when the lead prices are used as the instrument. Thus we do not use the lead price as the instrumental variable in this study.

## Results and discussion

### The effect of tobacco price on smoking participation

Table 1 presents the effect of cigarette price on daily smoking participation based on the regression Eq (1). The dependent variable is a dummy variable indicating daily smoking among men. It should be noted that binary models such as logit and probit, which treat the smoking status as a binary event, are often used when modelling tobacco participation. In this study, we use both linear probability regression and probit regression. The 2SLS estimators are consistently and widely used for the binary model with endogenous variables [29, 30]. S5 Table reports marginal effects from the probit regression. We used the results from the linear probability regression in Table 1 for interpretation.

**Table 1:** Regression of the smoking participation.

Explanatory variables	Daily cigarette smoking (yes = 1, no = 0)				Daily tobacco smoking (yes = 1, no = 0)			
	OLS	2SLS (one IV)	2SLS (two IVs)	2SLS (three IVs)	OLS	2SLS (one IV)	2SLS (two IVs)	2SLS (three IVs)
	(1)	(2)	(3)	(4)	(5)	(6)	(7)	(8)
Log of cigarette price	-0.0353***	-0.0799***	-0.0788**	-0.0861**	-0.0242***	-0.0433**	-0.0437**	-0.0464**
	(0.0093)	(0.0126)	(0.0355)	(0.0383)	(0.0079)	(0.0203)	(0.0214)	(0.0234)
Age	0.0266***	0.0265***	0.0264***	0.0261***	0.0373***	0.0372***	0.0371***	0.0367***
	(0.0014)	(0.0014)	(0.0014)	(0.0014)	(0.0017)	(0.0005)	(0.0005)	(0.0006)
Age squared	-0.0003***	-0.0003***	-0.0003***	-0.0003***	-0.0004***	-0.0004***	-0.0004***	-0.0004***
	(0.0000)	(0.0000)	(0.0000)	(0.0000)	(0.0000)	(0.0000)	(0.0000)	(0.0000)
Less than primary education	Reference							
Primary education	-0.0598***	-0.0595***	-0.0594***	-0.0598***	-0.0576***	-0.0576***	-0.0576***	-0.0581***
	(0.0208)	(0.0209)	(0.0211)	(0.0213)	(0.0217)	(0.0064)	(0.0068)	(0.0072)
Lower-secondary education	-0.1211***	-0.1211***	-0.1210***	-0.1214***	-0.1087***	-0.1087***	-0.1083***	-0.1084***
	(0.0206)	(0.0208)	(0.0206)	(0.0208)	(0.0219)	(0.0068)	(0.0071)	(0.0076)
Upper-secondary education	-0.1574***	-0.1571***	-0.1574***	-0.1579***	-0.1692***	-0.1690***	-0.1692***	-0.1696***
	(0.0226)	(0.0227)	(0.0229)	(0.0232)	(0.0237)	(0.0069)	(0.0073)	(0.0076)
College and above	-0.2004***	-0.1983***	-0.1962***	-0.1941***	-0.2345***	-0.2330***	-0.2313***	-0.2297***
	(0.0243)	(0.0246)	(0.0248)	(0.0252)	(0.0253)	(0.0067)	(0.0071)	(0.0075)
Kinh majority	0.0796***	0.0796***	0.0793***	0.0790***	0.0440**	0.0450***	0.0457***	0.0466***
	(0.0175)	(0.0175)	(0.0175)	(0.0177)	(0.0188)	(0.0066)	(0.0069)	(0.0073)
Wealth index	-0.0253***	-0.0256***	-0.0260***	-0.0266***	-0.0440***	-0.0442***	-0.0445***	-0.0452***
	(0.0078)	(0.0078)	(0.0078)	(0.0078)	(0.0081)	(0.0027)	(0.0028)	(0.0029)
Urban (urban = 1; rural = 0)	0.0650***	0.0646***	0.0643***	0.0639***	0.0442***	0.0443***	0.0444***	0.0445***
	(0.0141)	(0.0142)	(0.0142)	(0.0143)	(0.0144)	(0.0048)	(0.0050)	(0.0053)
Provincial overall CPI	-0.0245*	-0.0386***	-0.0378**	-0.0408**	-0.0150	-0.0210***	-0.0213***	-0.0234***
	(0.0131)	(0.0129)	(0.0184)	(0.0187)	(0.0124)	(0.0076)	(0.0079)	(0.0084)
Log of population density of provinces	0.0238	0.0341	0.0450	0.0669	-0.0369	-0.0374	-0.0390	-0.0274
	(0.0369)	(0.0392)	(0.0547)	(0.0629)	(0.0395)	(0.0266)	(0.0308)	(0.0358)
Log of per capita income of provinces	0.0037	0.0002	-0.0031	-0.0090	0.0152***	0.0100	0.0069	0.0023
	(0.0073)	(0.0056)	(0.0210)	(0.0212)	(0.0058)	(0.0111)	(0.0120)	(0.0130)
Province fixed-effects	Yes	Yes	Yes	Yes	Yes	Yes	Yes	Yes
Year fixed-effects	Yes	Yes	Yes	Yes	Yes	Yes	Yes	Yes
Constant	-0.0848	0.3557	0.2954	0.2690	0.1077	0.3475	0.3970	0.3888
	(0.2851)	(0.3399)	(0.6379)	(0.7130)	(0.2965)	(0.3144)	(0.3563)	(0.4055)
Observations	93,662	86,834	79,869	72,784	93,662	86,834	79,869	72,784
R-squared	0.103	n.a.	n.a.	n.a.	0.125	n.a.	n.a.	n.a.
Cragg-Donald Wald weak IV statistics		3.7e+04	1.5e+04	8561		3.7e+04	1.5e+04	8561
Hansen J statistic of overidentification test (P-value in parentheses)		n.a.	8561 (0.085)	3.338 (0.188)		n.a.	0.270 (0.603)	0.570 (0.752)

Note: These regressions use the sample of male individuals aged from 15.

The instrumental variables for log of cigarette price are one-year lag, two-year lag and three-year lag variables of log of cigarette prices.

Robust standard errors in parentheses. Standard errors are corrected for sampling weights and cluster correlation within individuals and clusters.

*** p<0.01,

** p<0.05,

* p<0.1.

R-squared is not reported for 2SLS.

Source: Estimation from GATS 2010 and 2015.

As mentioned in the estimation method section, we used information on age of smoking initiation and cessation to construct cohort data on smoking status from 2001 to 2015. The total number of individuals in the two GATS surveys used in the regression was 8,339. After being expanded over time, the total number of observations was 93,662. Since we expanded the data, there are no data on several time-variant explanatory variables, such as education and wealth index, for years other than 2010 and 2015. We used education and wealth index computed from the 2010 GATS as proxy measures of education and wealth index for years prior to 2010. For 2011 to 2015, we used education and wealth index computed from the 2015 GATS as proxy measures. In S6 Table, we ran regressions without controlling for education and wealth index. The results were very similar to those from regressions which controlled for these variables.

Table 1 reports results from OLS and 2SLS regression. In 2SLS, the instrumental variables for the log of current cigarette price are logs of lagged cigarette prices. When there is more than one instrument for the endogenous variable, a widely-used method to compute the instrumental estimates is two-stage least squares (2SLS). In the first stage, we regress the endogenous variable (the cigarette price in this study) on all of the control variables and the instruments and estimate the predicted values of the dependent variable. In the second stage, we regress the outcome variable (the cigarette smoking in this study) on the control variable and the predicted values of the endogenous variable from the first stage (see [25] for more detailed discussion). For comparison, we present 2SLS estimates using one instrument (columns 2 and 6), two instruments (columns 3 and 7) and three instruments (columns 4 and 8). The instruments are positively and strongly correlated with the current price. We performed Cragg-Donald and Kleibergen-Paap weak identification tests on the instruments and received the test statistics of over eight thousand (see Table 1). We use command ivreg2 in Stata to estimate the instrumental variable regression and conduct weak instrument and over-identification tests (see [28]). This result indicates extremely strong instruments (as a rule of thumb, if an F- statistic is under 10, the instruments might be weak [31]), which play an important role in reducing the bias in estimating the effect of the endogenous variable. Since the lagged variables are used as the instrument, the number of observations in the OLS regression is smaller than that in the 2SLS regression.

Using more than one instrument, we can conduct an over-identification test of the validity of the instruments [27, 28]. The joint null hypothesis under this test is that the instruments are valid instruments, i.e., they are uncorrelated with the error term. The last row of Table 1 shows the overidentification test statistics and the corresponding P-value in parentheses. It shows that the null hypothesis is not rejected at the 5% significance level. Estimates using different sets of instruments are very similar. For interpretation, we will use the estimate from using one instrument (columns 2 and 6 of Table 1), since this estimate is more efficient with a smaller standard error.

Both OLS and 2SLS regressions showed a negative effect of cigarette price on smoking. The point estimate from 2SLS was larger than that from OLS, but the difference was not statistically significant at the conventional level. The fact that the OLS point estimates are smaller than the 2SLS estimates suggests a possible downward bias in the OLS estimates. A possible reason is that the OLS estimates tend to reflect the interaction between the price and demand of cigarettes. As we know, an exogenous increase in the cigarette price leads to a decrease in the cigarette demand. However, the decrease in the cigarette demand can subsequently reduce the cigarette price. Thus the association between the cigarette price and the cigarette demand might be closer to zero than the causal effect of the cigarette price on the cigarette demand.

Column (2) of Table 1 shows that a one-percent increase in the cigarette price reduces the probability of cigarette smoking of individuals by 0.08 percentage points (the 95% CI from -0.06 to -0.10). In 2015, the daily smoking rate of men was 30.7%. A reduction of 0.08 percentage points is equal to 0.26% of the smoking rate. It means that the elasticity of the daily smoking prevalence of men with respect to the cigarette price was -0.26 (the 95% CI from -0.16% to -0.33%).

Most empirical studies estimate the price elasticity of demand for tobacco products. There is less empirical evidence on price elasticity of the smoking prevalence, especially in low-income countries. In Australia, Siahpush and colleagues [32] estimated the prevalence price elasticities for low-, medium-, and high-income groups at −0.32, −0.04, and −0.02, respectively. According to Sharbaugh and colleagues, an additional $0.25 per-pack tax was associated with a 0.6 percentage point reduction in the smoking prevalence in the United States [33]. Using the average price and the smoking prevalence, we can compute the prevalence of smoking elasticity to the price at 0.95. Compared with these two studies, the price elasticity of the smoking prevalence for Vietnam is slightly lower. Possibly, the cigarette price is rather low in Vietnam, and tobacco products remain affordable by a large number of people.

In addition to the cigarette smoking, we also estimated the effect of the cigarette price on daily tobacco smoking. i.e., the dependent variable is measured by smoking of any tobacco products. Columns from (5) to (8) of Table 1 show that the effect of the cigarette price on tobacco smoking was also negative and statistically significant. According to 2SLS regression in column (6), a one-percent increase in the cigarette price resulted in a 0.043 percentage point decrease in the probability of tobacco smoking, equivalent to 0.11% of the tobacco smoking rate. The point estimates of the effect of cigarette price on tobacco smoking was smaller than those on cigarette smoking, since tobacco products include not only cigarettes, but also other products such as traditional and shisha water-pipes, smokeless tobacco, and these non-cigarette products might be less sensitive to cigarette price.

**Fig 3:**
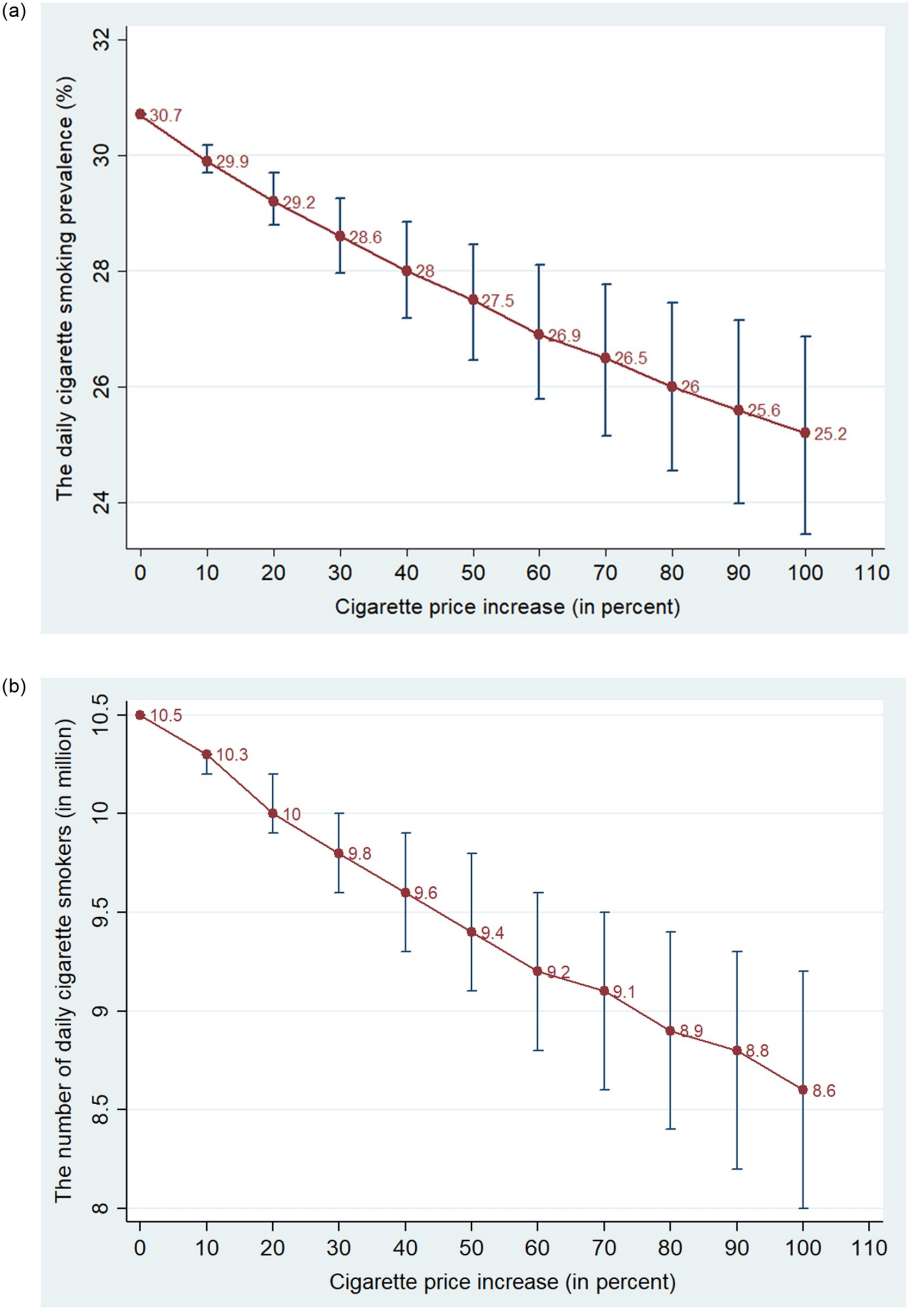
Simulated effects of the price increase on daily male cigarette smoking. Panel A. The daily male cigarette smoking rate (%) corresponding the percentage change in the price. Panel B. The number of daily male smokers (million) corresponding the percentage change in the price. Note: This figure presents the estimated prevalence and its 95 confidence intervals of daily cigarette smoking among men (in percent) and the number of males with daily cigarette smoking (in million) corresponding to the increase in the cigarette price. Source: Estimation from GATS 2010 and 2015.

To examine how cigarette smoking among men changes as a result of a price increase, we simulated the effect of the cigarette price on the prevalence of cigarette smoking and the number of male cigarette smokers. Using the estimated effect of the log of cigarette price on the cigarette smoking participation at -0.0799 (column 2 in Table 1), we predicted how smoking prevalence changes as a result of a change in the cigarette price. The daily cigarette smoking rate in 2015 was 30.7%. The national average price of a Vinataba pack was 20.6 thousand VND in 2015. If this price is increased by 10%, the cigarette smoking prevalence was predicted to decrease from 30.7% to 29.9% (Panel A of Fig 3). It is estimated that the number of cigarettes declines from 10.53 to 10.26 million (Panel B of Fig 3), a reduction of 270 thousand smokers. A larger increase in the cigarette price led to a larger decrease in smoking prevalence and the number of smokers. If the cigarette price is increased by 100%, it is predicted that the cigarette smoking rate will decrease by 5.5 percentage points and the number of cigarette smokers will decline by 1.89 million.

**Fig 4:**
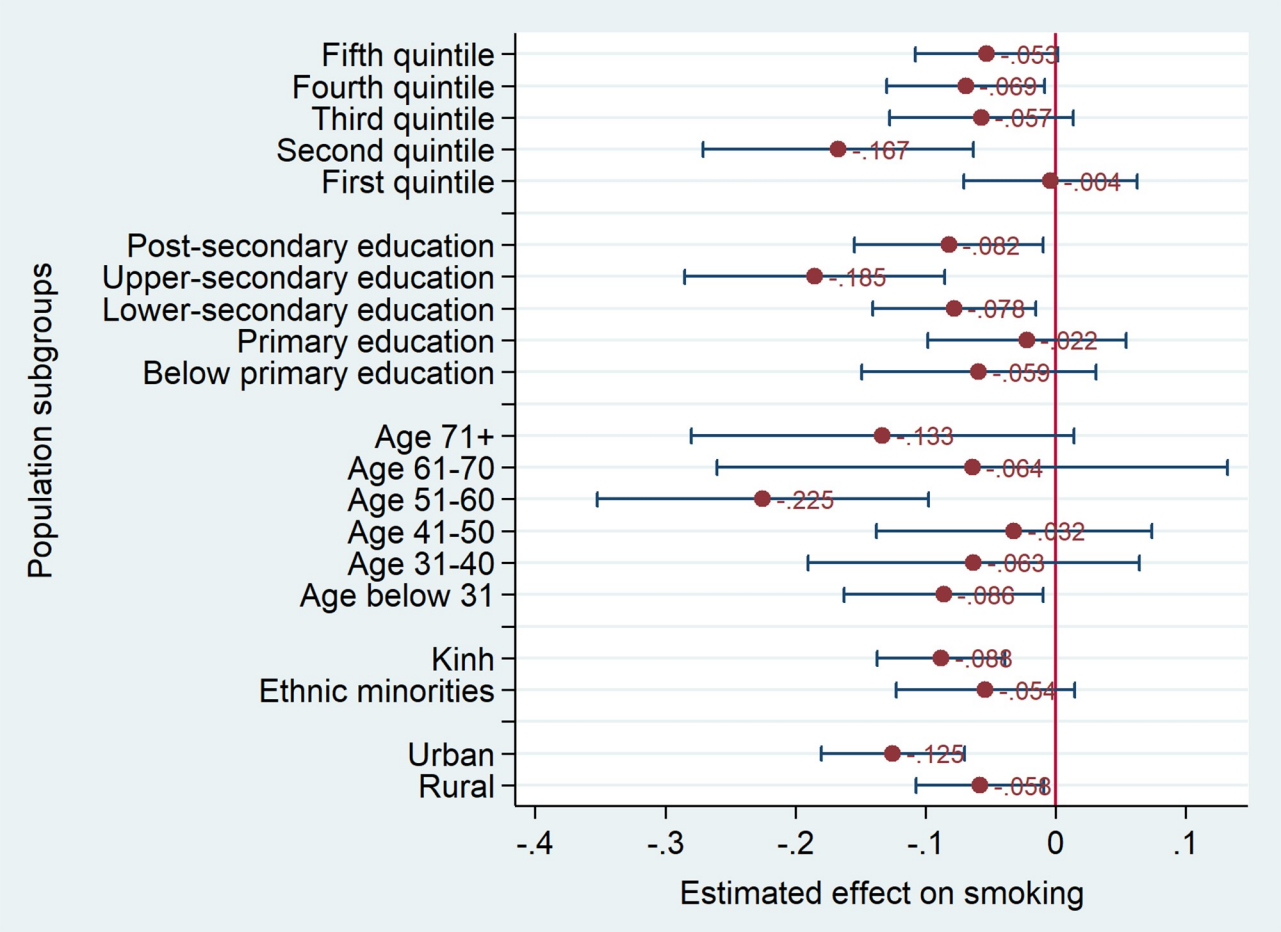
Heterogeneous effect of the cigarette prices on the smoking probability. Source: Estimation from GATS 2010 and 2015.

We used the same model specification as column 2 in Table 1 to estimate the effect of log of the cigarette price on cigarette smoking for different population sub-groups. Fig 4 presents the point estimates and their 95% confidence interval of the log of the cigarette price on the cigarette smoking. All the point estimates were negative. However, the effect was not statistically significant for some groups. By wealth quintile, the effect of the cigarette price on smoking was largest for people in the second wealth quintile. The effect of the cigarette price was small and not statistically significant for people in the lowest wealth quintile. It could be explained by the availability of variety of cigarette brands in Vietnam market [34], and the low wealth quintiles tend to use the economy brands. Another possible reason for lower elasticity of the smoking prevalence with respect to the cigarette price among the disadvantaged groups is that these groups are more likely to use homemade tobacco products which can substitute for cigarettes. The effect tended to be higher for urban and Kinh people compared to rural and ethnic minority people, though the difference in the effect between these groups was not statistically significant.

The effect of cigarette price tended to be larger on people with upper-secondary education compared to people with other levels of education. By age groups, the effect of the cigarette price was significant for people below 31 years of age and those aged 51–60. The significant effect observed for young people implies that higher cigarette prices might delay smoking onset. This is consistent with findings from Guindon [16] and Nguyen and colleagues [17] on the smoking-onset delaying effect of cigarette prices.

Table 1 also reveals several interesting findings regarding the effect of different variables affecting smoking. We found there to be an inverted-U shaped relation between smoking and age. The probability of smoking increased with age, but after achieving a peak at around age 47 it started to decrease. People with higher education levels were less likely to smoke than those with lower education levels. There was a negative association between smoking and wealth index, indicating a lower smoking rate among wealthier people. However, urban and Kinh people had a higher probability of smoking compared to rural and ethnic minority people.

### The effect of tobacco price on tobacco consumption expenditure

This section reports and discusses the estimation of the cigarette price on tobacco expenditure among households in Vietnam. Table 2 reports the regression of the log of per capita tobacco expenditure on log of cigarette prices. We used 2SLS results for interpretation. The instrumental variable for the cigarette price is the lagged price (in the previous year). The lagged price is very strongly and positively correlated with the current price. The test statistics from Cragg-Donald and Kleibergen-Paap weak identification tests were higher than ten thousand, indicating a very strong instrument (as a rule of thumb, if an F-statistic is under 10, the instruments may be weak [31]).

**Table 2:** Regression of the log of per capita tobacco consumption expenditure.

Explanatory variables	Log of per capita tobacco consumption expenditure (including home production)				Log of per capita tobacco consumption expenditure (excluding home production)			
	OLS	2SLS (one IV)	2SLS (two IVs)	2SLS (three IVs)	OLS	2SLS (one IV)	2SLS (two IVs)	2SLS (three IVs)
	(1)	(2)	(3)	(4)	(5)	(6)	(7)	(8)
Log of cigarette price	-0.1347	-0.4255**	-0.3743*	-0.3748*	-0.1274	-0.4018**	-0.3515*	-0.3529*
	(0.1202)	(0.2021)	(0.1976)	(0.1972)	(0.1219)	(0.2040)	(0.1995)	(0.1990)
Household size	-0.0776***	-0.0777***	-0.0776***	-0.0776***	-0.0789***	-0.0789***	-0.0789***	-0.0789***
	(0.0066)	(0.0066)	(0.0066)	(0.0066)	(0.0066)	(0.0066)	(0.0066)	(0.0066)
Proportion of children below 15	-0.3209***	-0.3193***	-0.3196***	-0.3196***	-0.3072***	-0.3058***	-0.3060***	-0.3060***
	(0.0559)	(0.0558)	(0.0558)	(0.0558)	(0.0563)	(0.0563)	(0.0563)	(0.0563)
Proportion of older people from 60	-0.2735***	-0.2736***	-0.2736***	-0.2736***	-0.2571***	-0.2577***	-0.2576***	-0.2576***
	(0.0555)	(0.0555)	(0.0555)	(0.0555)	(0.0568)	(0.0568)	(0.0568)	(0.0568)
Proportion of female members	-0.6596***	-0.6586***	-0.6588***	-0.6588***	-0.6571***	-0.6561***	-0.6563***	-0.6563***
	(0.0512)	(0.0512)	(0.0512)	(0.0512)	(0.0516)	(0.0516)	(0.0516)	(0.0516)
Kinh majority	0.0188	0.0180	0.0181	0.0181	0.0303	0.0295	0.0296	0.0296
	(0.0336)	(0.0336)	(0.0336)	(0.0336)	(0.0338)	(0.0338)	(0.0338)	(0.0338)
Male household head	0.2236***	0.2234***	0.2234***	0.2234***	0.2225***	0.2222***	0.2223***	0.2223***
	(0.0247)	(0.0247)	(0.0247)	(0.0247)	(0.0250)	(0.0249)	(0.0249)	(0.0249)
Age of household head	-0.0066	-0.0065	-0.0065	-0.0065	-0.0054	-0.0054	-0.0054	-0.0054
	(0.0048)	(0.0048)	(0.0048)	(0.0048)	(0.0049)	(0.0049)	(0.0049)	(0.0049)
Squared age of household head	0.0000	0.0000	0.0000	0.0000	0.0000	0.0000	0.0000	0.0000
	(0.0000)	(0.0000)	(0.0000)	(0.0000)	(0.0000)	(0.0000)	(0.0000)	(0.0000)
Head less than primary degree	Reference							
Head with primary education	-0.1079***	-0.1078***	-0.1079***	-0.1079***	-0.1025***	-0.1024***	-0.1024***	-0.1024***
	(0.0242)	(0.0242)	(0.0242)	(0.0242)	(0.0244)	(0.0244)	(0.0244)	(0.0244)
Head with lower-secondary education	-0.1838***	-0.1842***	-0.1841***	-0.1841***	-0.1806***	-0.1810***	-0.1809***	-0.1809***
	(0.0274)	(0.0274)	(0.0274)	(0.0274)	(0.0276)	(0.0276)	(0.0276)	(0.0276)
Head with upper-secondary education	-0.1870***	-0.1872***	-0.1871***	-0.1871***	-0.1814***	-0.1815***	-0.1814***	-0.1814***
	(0.0314)	(0.0314)	(0.0314)	(0.0314)	(0.0317)	(0.0317)	(0.0317)	(0.0317)
Head with post-secondary education	-0.5850***	-0.5852***	-0.5852***	-0.5852***	-0.5834***	-0.5837***	-0.5836***	-0.5836***
	(0.0558)	(0.0558)	(0.0558)	(0.0558)	(0.0563)	(0.0562)	(0.0562)	(0.0562)
Log of per capita income	0.3469***	0.3473***	0.3473***	0.3473***	0.3470***	0.3475***	0.3474***	0.3474***
	(0.0165)	(0.0165)	(0.0165)	(0.0165)	(0.0166)	(0.0166)	(0.0166)	(0.0166)
Urban (urban = 1; rural = 0)	0.1066***	0.1059***	0.1060***	0.1060***	0.1138***	0.1131***	0.1132***	0.1132***
	(0.0256)	(0.0256)	(0.0256)	(0.0256)	(0.0258)	(0.0258)	(0.0258)	(0.0258)
Provincial overall CPI	-0.0464	-0.1306	-0.1158	-0.1160	-0.0414	-0.1207	-0.1061	-0.1065
	(0.0676)	(0.0812)	(0.0803)	(0.0802)	(0.0678)	(0.0814)	(0.0806)	(0.0805)
Log of population density of provinces	-0.4389	-0.3950	-0.4027	-0.4026	-0.4397	-0.3983	-0.4059	-0.4056
	(0.2860)	(0.2864)	(0.2863)	(0.2864)	(0.2866)	(0.2869)	(0.2869)	(0.2869)
Log of per capita income of provinces	-0.0016	-0.0186	-0.0157	-0.0157	0.0104	-0.0057	-0.0028	-0.0028
	(0.1270)	(0.1271)	(0.1271)	(0.1271)	(0.1279)	(0.1280)	(0.1281)	(0.1281)
Province fixed-effects	Yes	Yes	Yes	Yes	Yes	Yes	Yes	Yes
Year fixed-effects	Yes	Yes	Yes	Yes	Yes	Yes	Yes	Yes
Constant	6.5391**	9.2123***	8.7422***	8.7464***	6.2919**	8.8138***	8.3517***	8.3644***
	(2.7475)	(3.1472)	(3.1249)	(3.1197)	(2.7678)	(3.1721)	(3.1507)	(3.1451)
Observations	39,061	39,061	39,061	39,061	38,772	38,772	38,772	38,772
R-squared	0.254	n.a.	n.a.	n.a.	0.254	n.a.	n.a.	n.a.
Cragg-Donald Wald weak IV statistics		2.0e+04	1.0e+04	7017		2.0e+04	1.0e+04	7017
Hansen J statistic of overidentification test (P-value in parentheses)			3.052 (0.081)	3.061 (0.217)			2.939 (0.089)	2.969 (0.227)

Note: These regressions use the sample of households. The instrumental variables for log of cigarette price are one-year lag, two-year lag and three-year lag variables of log of cigarette prices.

R-squared is not reported in 2SLS.

Robust standard errors in parentheses. Standard errors are corrected for sampling weights and cluster correlation within clusters.

*** p<0.01,

** p<0.05,

* p<0.1.

Source: Estimation from VHLSSs 2006–2016.

There are two dependent variables. The first is the log of total expenditure of all tobacco products, including homemade tobacco products (reported in columns 1 to 4 of Table 2), and the second is the log of purchased tobacco products (reported in columns 5 to 8 of Table 2). The effect of cigarette prices was very similar in regressions of the two dependent variables. Similar to Table 1, the OLS point estimates are smaller than the 2SLS estimates, suggesting a possible downward bias in the OLS estimates (though the differences between the OLS and 2SLS estimates are not statistically significant). We use the 2SLS estimates for interpretation. Our estimate of the price elasticity of tobacco expenditure is consistent with previous studies. It is quite close to the estimate from Eozenou and Fishburn [14], which estimated the price elasticity for cigarette demand at around -0.53, using Vietnam Living Standard Survey 1998. Our estimated elasticity is similar to those found in Latin American countries, where own-price elasticity for cigarettes was estimated at -0.4 [9]. A meta-analysis of elasticities from Gallet and List [35] estimates an average price elasticity of –0.48 based on 86 studies. Jha and Chaloupka [36] found the price elasticity of tobacco expenditure to be approximately -4% in high-income countries and approximately -8% in low-income countries. Chaloupka and colleagues [6] also concluded that tobacco demand is more elastic to the price in low-income countries compared to high-income countries.

**Fig 5:**
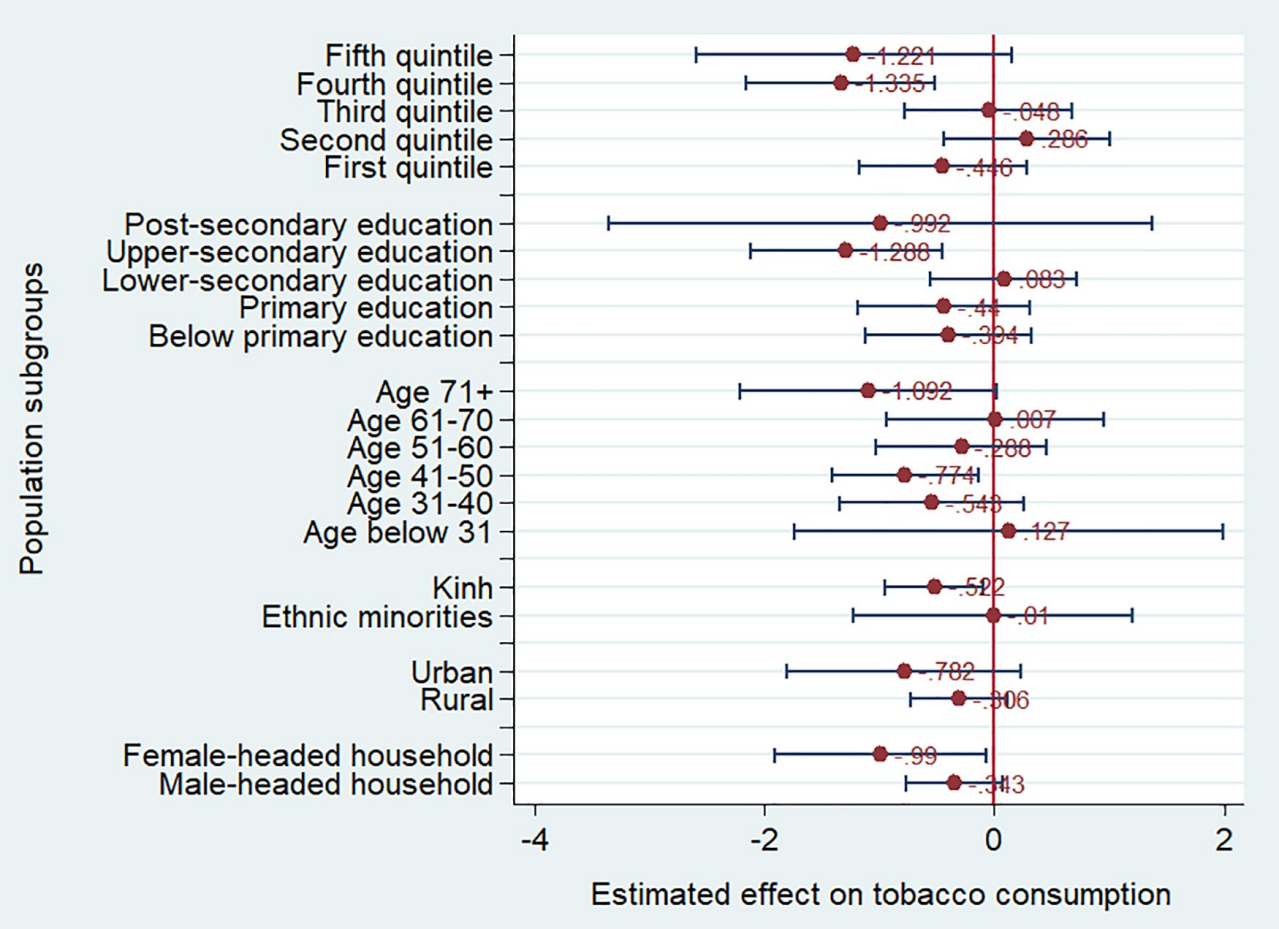
Heterogeneous effect of cigarette prices on the tobacco expenditure. Source: Estimation from VHLSSs 2006–2016.

We used a similar model specification as column 6 in Table 2 to estimate the elasticity of tobacco expenditure with respect to prices among different groups of households. Fig 5 shows that the price elasticity of tobacco expenditure was higher for households in higher income quintiles. The price elasticity was highest among households with household heads who have obtained upper-secondary education. This finding is consistent with our results on the highest effect of cigarette prices on the smoking participation among men with upper-secondary education (Fig 4). We also found cigarette price to have a larger effect on tobacco expenditure among urban and Kinh households, compared to rural and ethnic minority households.

Finally, Table 2 shows several important findings on the association between tobacco expenditure and household characteristics. Households with more members had lower per capita tobacco expenditure compared to households with fewer members. We found that households with more children and older people spend less on tobacco. The amount of tobacco expenditure among urban households was approximately 11 percent higher than that of rural households. This is consistent with findings from Table 1 which indicate that urban people had a higher smoking rate compared to their rural counterparts. As expected, male-headed households were much more likely to consume tobacco than households headed by female. This is likely because smoking in Vietnam is mainly prevalent among men. In female-headed households, household heads are more likely to be widowed, divorced, or have migrating husbands.

We found a strong correlation between education of household heads and tobacco expenditure. The tobacco expenditure was much lower in households with highly-educated heads than in households with lowly-educated heads. Higher income was associated with higher tobacco expenditure. The income elasticity of tobacco expenditure was estimated at 0.35 (column 4), suggesting that tobacco is a normal good. As per capita income increased by 1%, the demand for tobacco increased by around 0.35%. This estimate is very similar to the estimates of income elasticity of tobacco demand at 0.3 to 0.4 in other studies such as Gallet and List (2003) [35], Martinez and colleagues (2015) [37], Álvarez and colleagues (2020) [38].

It should be noted that Table 1 indicates that people in lower wealth quintiles are more likely to smoke than those in higher wealth quintiles, while Table 2 shows a positive correlation between income and expenditure on tobaccos of households. Our finding from Table 1 is consistent with a well-known evidence that the poor tends to smoke more than the rich in low- and middle-income countries [39]. For the case of Vietnam, Kien and colleagues (2017) also find the proportion of adult male hardcore smokers is higher among the poor than among the rich [40]. However, in Table 2, We estimate the elasticity of tobacco consumption of households with respect to the current income. Put it differently, we limit the sample to households with positive tobacco consumption. Our findings on income elasticity is also consistent with previous studies which suggest that tobacco is a normal good with positive income elasticity.

## Conclusions

In this study we examined the effect of cigarette price on smoking participation among adult men and tobacco spending among households in Vietnam. Using regression analyses, we found that a one-percent increase in the real cigarette price reduces the probability of cigarette smoking of males by 0.08 percentage points (the 95% CI from -0.06 to -0.10), equivalent to the price elasticity of smoking prevalence at -0.26 (the 95% CI from -0.16% to -0.33%). When the cigarette price is increased by 10%, the cigarette smoking prevalence among men decreased from 30.7% to 29.9% and the number of male smokers declined by 270 thousand. Higher cigarette prices also reduce tobacco consumption expenditure among households. A one-percent increase in the price leds to a 0.4 percent decrease in per capita tobacco expenditure of households.

Findings from this study suggest that increasing tobacco taxation can be an effective measure to reduce smoking in Vietnam. The government should increase tobacco tax to reduce the smoking prevalence and its expenditure. The current tax rate of Vietnam is still very low, account for about one-third of the retail price [41] while the World Health Organization recommends that the tax share should represent at least 75% of the retail price. In addition, Viet Nam is currently using only an ad valorem tax, calculated as a percentage of the factory price. This tax system has a shortcoming that it encourages the production as well as consumption of low-cost cigarettes to avoid tax payment. This mitigates the effectiveness of tax increases in reducing cigarette consumption. For tax policy to be effective, tobacco tax needs to increase sharply to meet the WHO’ recommendation on tax burden and to increase continuously in consideration to the inflation rate.

The heterogeneous analysis shows a lesser effect of cigarette prices on cigarette smoking and tobacco consumption for more disadvantaged groups, such as lower-income, ethnic minority, and rural households. Low-income people are more likely to use cheap and homemade products, which are less sensitive to cigarette prices. Moreover, access to illicit cigarettes can also erode the effectiveness of cigarette taxation policies. Nguyen and colleagues) found that illicit cigarettes accounted for 20.7% of total cigarette consumption in Vietnam in 2012 [42]. Thus it is also very important to strong measures to reduce and prevent illicit tobacco trade in Vietnam.

Finally, provision of information and health education on harmful effects of tobacco use is an important dissemination policy to reduce smoking among the disadvantaged groups. Strict enforcement of regulations on banning tobacco advertising and promotion can also reduce the smoking onset and help smoker to quit smoking or reduce consumption, especially among teens and young people.

## Supporting information

S1 Fig Cigarette price. (TIF)

S2 Fig Vinataba price and tobacco CPI. (TIF)

S1 Table The smoking rate of men by socio-economic characteristics (%). (DOCX)

S2 Table Tobacco consumption expenditure of households. (DOCX)

S3 Table Definition and summary statistics of explanatory variables in regression of smoking participation. (DOCX)

S4 Table Definition and summary statistics of explanatory variables in the regression of tobacco consumption expenditure of households. (DOCX)

S5 Table Probit regression of the smoking participation. (DOCX)

S6 Table Regression of the smoking participation without time-variant control variables. (DOCX)

S1 Data Expanded GAT data and VHLSS data. (RAR)
