# Supplementary figures and images for: The impact of cigarette prices on smoking participation and tobacco expenditure in Vietnam
Source: PLoS One. 2021 Dec 14;16(12):e0260415. doi: 10.1371/journal.pone.0260415 (PMC8670683; doi:10.1371/journal.pone.0260415)

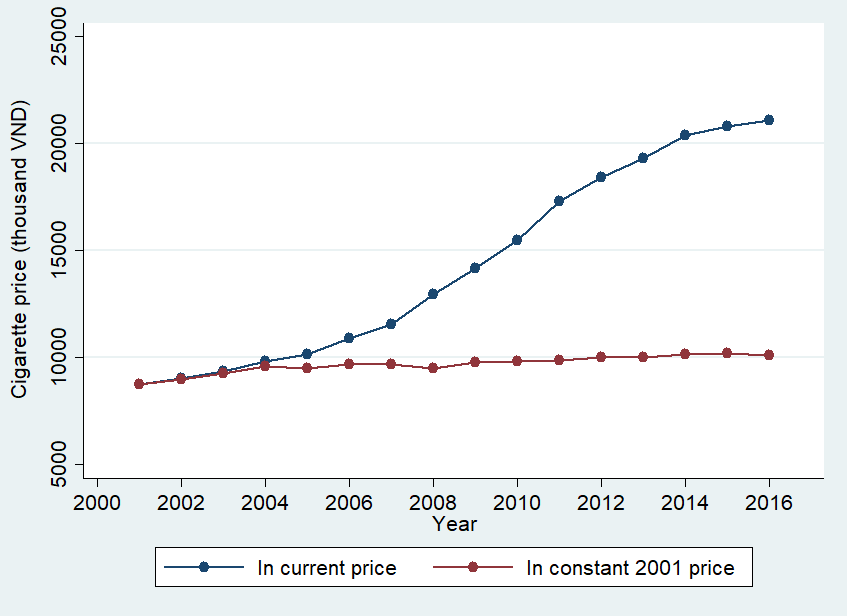

Supplement: S1 Fig — (TIF) [file pone.0260415.s001.tif]

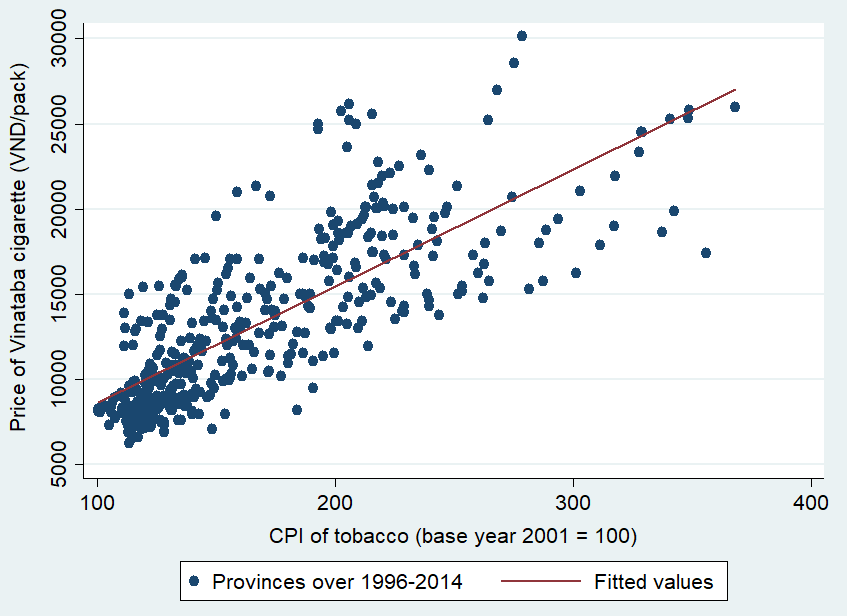

Supplement: S2 Fig — (TIF) [file pone.0260415.s002.tif]
