# Supplementary material for: The impact of cigarette prices on smoking participation and tobacco expenditure in Vietnam
Source: PLoS One. 2021 Dec 14;16(12):e0260415. doi: 10.1371/journal.pone.0260415 (PMC8670683; doi:10.1371/journal.pone.0260415)
Supplement: S1 Table — (DOCX) [file pone.0260415.s003.docx]

**S1 Table. The smoking rate of men by socio-economic characteristics (%).**

| Population subgroups | The tobacco smoking rate (%) | | The daily tobacco smoking rate (%) | | The daily cigarette smoking rate (%) | |
| --- | --- | --- | --- | --- | --- | --- |
|  | GATS 2010 | GATS 2015 | GATS 2010 | GATS 2015 | GATS 2010 | GATS 2015 |
| Total | 47.4 | 45.3 | 38.7 | 38.7 | 31.3 | 30.7 |
| ***Areas*** |  |  |  |  |  |  |
| Rural | 47.3 | 46.7 | 38.6 | 40.3 | 28.9 | 30.0 |
| Urban | 47.7 | 42.7 | 39.1 | 35.7 | 36.7 | 32.2 |
| ***Ethnic and Kinh*** |  |  |  |  |  |  |
| Ethnic minorities | 51.8 | 45.8 | 40.1 | 38.3 | 27.3 | 25.1 |
| Kinh | 46.5 | 45.2 | 38.5 | 38.8 | 32.1 | 31.9 |
| ***Age of household head*** |  |  |  |  |  |  |
| 30 and less | 33.0 | 30.1 | 24.7 | 24.3 | 22.0 | 21.3 |
| 31-40 | 61.3 | 54.2 | 48.6 | 44.9 | 41.9 | 38.2 |
| 41-50 | 59.1 | 60.1 | 52.0 | 52.4 | 39.1 | 39.6 |
| 51-60 | 63.4 | 55.7 | 54.9 | 50.2 | 42.7 | 37.6 |
| 61-70 | 44.5 | 46.3 | 39.3 | 42.0 | 23.9 | 27.1 |
| 71+ | 25.5 | 27.6 | 19.9 | 25.4 | 12.5 | 16.9 |
| ***Education of household head*** |  |  |  |  |  |  |
| < Primary | 59.4 | 58.7 | 53.5 | 53.8 | 42.3 | 42.0 |
| Primary | 53.5 | 56.9 | 44.2 | 49.0 | 36.4 | 40.8 |
| Lower-secondary | 42.4 | 43.0 | 34.3 | 37.7 | 25.9 | 27.3 |
| Upper-secondary | 40.9 | 37.2 | 30.4 | 30.1 | 26.4 | 24.1 |
| Post-secondary | 38.4 | 34.1 | 27.6 | 25.7 | 25.2 | 23.8 |
| ***Expenditure quintiles*** |  |  |  |  |  |  |
| Poorest | 52.6 | 54.6 | 43.8 | 47.9 | 32.9 | 36.0 |
| Near poorest | 54.8 | 49.8 | 43.6 | 43.0 | 29.5 | 33.3 |
| Middle | 49.8 | 46.0 | 41.9 | 40.9 | 34.8 | 30.5 |
| Near richest | 43.4 | 39.5 | 34.5 | 31.5 | 29.6 | 26.9 |
| Richest | 37.3 | 35.3 | 29.5 | 29.1 | 27.7 | 26.1 |

Source: Estimation from GATS 2010 and 2015.
