# Supplementary material for: The impact of cigarette prices on smoking participation and tobacco expenditure in Vietnam
Source: PLoS One. 2021 Dec 14;16(12):e0260415. doi: 10.1371/journal.pone.0260415 (PMC8670683; doi:10.1371/journal.pone.0260415)
Supplement: S2 Table — (DOCX) [file pone.0260415.s004.docx]

**S2 Table. Tobacco consumption expenditure of households.**

| Population subgroups | The share of tobacco consumption in total consumption expenditure (%) | | Per capita tobacco consumption expenditure (thousand VND) | |
| --- | --- | --- | --- | --- |
|  | VHLSS 2006 | VHLSS 2016 | VHLSS 2006 | VHLSS 2016 |
| Total | 1.41 | 0.78 | 188.3 | 220.6 |
| ***Areas*** |  |  |  |  |
| Rural | 1.42 | 0.84 | 148.5 | 194.0 |
| Urban | 1.38 | 0.64 | 270.1 | 251.4 |
| ***Ethnic and Kinh*** |  |  |  |  |
| Ethnic minorities | 1.46 | 0.83 | 110.5 | 125.6 |
| Kinh | 1.40 | 0.76 | 193.0 | 229.9 |
| ***Gender of household head*** |  |  |  |  |
| Male | 1.45 | 0.82 | 179.2 | 222.5 |
| Female | 1.27 | 0.62 | 187.1 | 175.7 |
| ***Age of household head*** |  |  |  |  |
| 30 and less | 1.45 | 0.91 | 137.3 | 178.3 |
| 31-40 | 1.53 | 0.83 | 177.7 | 229.7 |
| 41-50 | 1.36 | 0.81 | 186.1 | 226.6 |
| 51-60 | 1.40 | 0.74 | 198.2 | 218.0 |
| 61-70 | 1.35 | 0.74 | 170.1 | 189.5 |
| 71+ | 1.33 | 0.65 | 161.6 | 162.1 |
| ***Education of household head*** |  |  |  |  |
| < Primary | 1.85 | 1.19 | 187.8 | 241.7 |
| Primary | 1.62 | 0.97 | 189.5 | 237.7 |
| Lower-secondary | 1.09 | 0.54 | 144.6 | 157.7 |
| Upper-secondary | 1.07 | 0.58 | 201.8 | 238.7 |
| Post-secondary | 0.80 | 0.33 | 227.9 | 182.9 |
| ***Wealth index quintiles*** |  |  |  |  |
| Poorest | 1.50 | 1.02 | 81.7 | 120.8 |
| Near poorest | 1.49 | 0.93 | 126.6 | 192.1 |
| Middle | 1.52 | 0.79 | 174.6 | 226.6 |
| Near richest | 1.36 | 0.63 | 221.2 | 257.0 |
| Richest | 1.12 | 0.40 | 342.4 | 297.0 |

Source: Authors’ estimation from VHLSSs.
