# Supplementary material for: The impact of cigarette prices on smoking participation and tobacco expenditure in Vietnam
Source: PLoS One. 2021 Dec 14;16(12):e0260415. doi: 10.1371/journal.pone.0260415 (PMC8670683; doi:10.1371/journal.pone.0260415)
Supplement: S3 Table — (DOCX) [file pone.0260415.s005.docx]

**S3 Table. Definition and summary statistics of explanatory variables in regression of smoking participation.**

| Variables | Type | Level | Source | GATS 2010 | | GATS 2015 | |
| --- | --- | --- | --- | --- | --- | --- | --- |
|  |  |  |  | Mean | Std. Dev. | Mean | Std. Dev. |
| Age of individuals | Discrete | Individuals | GATS | 37.67 | 16.47 | 38.87 | 16.49 |
| Individuals completed less than primary education | Binary | Individuals | GATS | 0.161 | 0.368 | 0.136 | 0.343 |
| Individuals completed primary education | Binary | Individuals | GATS | 0.253 | 0.435 | 0.195 | 0.396 |
| Individuals completed lower-secondary education | Binary | Individuals | GATS | 0.337 | 0.473 | 0.319 | 0.466 |
| Individuals completed upper-secondary education | Binary | Individuals | GATS | 0.184 | 0.388 | 0.192 | 0.394 |
| Individuals completed college or above | Binary | Individuals | GATS | 0.065 | 0.247 | 0.158 | 0.365 |
| Ethnicity of individuals (Kinh=1, ethnic minorities=0) | Binary | Individuals | GATS | 0.835 | 0.371 | 0.831 | 0.375 |
| Wealth index of individuals | Continuous | Individuals | GATS | 0.007 | 0.996 | 0.020 | 1.007 |
| Individuals living in urban areas (urban=1; rural=0) | Binary | Individuals | GATS | 0.301 | 0.459 | 0.331 | 0.471 |
| Province-level overall CPI (base year is 2001 with CPI=1) | Continuous | Province | GSO | 1.620 | 0.217 | 2.097 | 0.424 |
| Log of cigarette price (measured by Vinataba cigarette price) | Continuous | Province | GSO | 9.113 | 0.209 | 9.221 | 0.248 |
| Log of lagged cigarette price (measured by Vinataba cigarette price) | Continuous | Province | GSO | 9.090 | 0.226 | 9.224 | 0.241 |
| Log of population density of provinces | Continuous | Province | GSO | 7.011 | 0.502 | 7.482 | 0.738 |
| Log of per capita income of provinces | Continuous | Province | GSO | 9.525 | 0.344 | 10.330 | 0.316 |

Source: Estimation from GATS 2010 and 2015.
