# Supplementary material for: The impact of cigarette prices on smoking participation and tobacco expenditure in Vietnam
Source: PLoS One. 2021 Dec 14;16(12):e0260415. doi: 10.1371/journal.pone.0260415 (PMC8670683; doi:10.1371/journal.pone.0260415)
Supplement: S4 Table — (DOCX) [file pone.0260415.s006.docx]

**S4 Table. Definition and summary statistics of explanatory variables in the regression of tobacco consumption expenditure of households.**

| Variables | Type | Level | Source | VHLSS 2006 | | VHLSS 2016 | |
| --- | --- | --- | --- | --- | --- | --- | --- |
|  |  |  |  | Mean | Std. Dev. | Mean | Std. Dev. |
| The number of members in household | Discrete | Household | VHLSS | 4.376 | 1.631 | 4.075 | 1.544 |
| Proportion of children (age below 15) in household | Continuous | Household | VHLSS | 0.219 | 0.206 | 0.209 | 0.199 |
| Proportion of older members (age 60+) in household | Continuous | Household | VHLSS | 0.115 | 0.235 | 0.124 | 0.241 |
| Proportion of female members (age 60+) in household | Continuous | Household | VHLSS | 0.500 | 0.180 | 0.485 | 0.178 |
| Ethnicity of household heads (Kinh=1, ethnic minorities=0) | Binary | Household | VHLSS | 0.870 | 0.336 | 0.836 | 0.370 |
| Household living in urban areas (urban=1; rural=0) | Binary | Household | VHLSS | 0.257 | 0.437 | 0.282 | 0.450 |
| Gender of household head (male=1; female=0) | Binary | Household | VHLSS | 0.791 | 0.406 | 0.826 | 0.379 |
| Age of household head | Discrete | Household | VHLSS | 49.15 | 13.13 | 50.86 | 12.73 |
| Household head completed less than primary education | Binary | Household | VHLSS | 0.250 | 0.433 | 0.213 | 0.409 |
| Household head completed primary education | Binary | Household | VHLSS | 0.248 | 0.432 | 0.247 | 0.431 |
| Household head completed lower-secondary education | Binary | Household | VHLSS | 0.274 | 0.446 | 0.278 | 0.448 |
| Household head completed upper-secondary education | Binary | Household | VHLSS | 0.184 | 0.388 | 0.193 | 0.395 |
| Household head completed college or above | Binary | Household | VHLSS | 0.043 | 0.204 | 0.069 | 0.253 |
| Log of per capita income of households | Continuous | Household | VHLSS | 9.608 | 0.662 | 10.249 | 0.717 |
| Province-level overall CPI (base year is 2001 with CPI=1) | Continuous | Province | GSO | 1.050 | 0.031 | 2.007 | 0.488 |
| Log of cigarette price (measured by Vinataba cigarette price) | Continuous | Province | GSO | 9.218 | 0.223 | 9.280 | 0.267 |
| Log of lagged cigarette price (measured by Vinataba cigarette price) | Continuous | Province | GSO | 9.198 | 0.235 | 9.281 | 0.261 |
| Log of population density of provinces | Continuous | Province | GSO | 6.002 | 1.014 | 6.084 | 1.070 |
| Log of per capita income of provinces | Continuous | Province | GSO | 9.676 | 0.266 | 10.394 | 0.312 |

Source: Authors’ estimation from VHLSSs.
