# Supplementary material for: The impact of cigarette prices on smoking participation and tobacco expenditure in Vietnam
Source: PLoS One. 2021 Dec 14;16(12):e0260415. doi: 10.1371/journal.pone.0260415 (PMC8670683; doi:10.1371/journal.pone.0260415)
Supplement: S5 Table — (DOCX) [file pone.0260415.s007.docx]

**S5 Table. Probit regression of the smoking participation.**

| Explanatory variables | Cigarette smoking  (yes=1, no=0) | | Tobacco smoking  (yes=1, no=0) | |
| --- | --- | --- | --- | --- |
|  | Probit | IV-Probit | Probit | IV-Probit |
| Log of cigarette price | -0.0328*** | -0.0762*** | -0.0258*** | -0.0486*** |
|  | (0.0095) | (0.0138) | (0.0079) | (0.0107) |
| Age | 0.0300*** | 0.0299*** | 0.0398*** | 0.0396*** |
|  | (0.0018) | (0.0018) | (0.0022) | (0.0020) |
| Age squared | -0.0004*** | -0.0004*** | -0.0004*** | -0.0004*** |
|  | (0.0000) | (0.0000) | (0.0000) | (0.0000) |
| Less than primary education |  |  | Reference |  |
| Primary education | -0.0543*** | -0.0540*** | -0.0523*** | -0.0523** |
|  | (0.0190) | (0.0192) | (0.0203) | (0.0206) |
| Lower-secondary education | -0.1098*** | -0.1097*** | -0.1005*** | -0.1003*** |
|  | (0.0193) | (0.0194) | (0.0204) | (0.0208) |
| Upper-secondary education | -0.1478*** | -0.1476*** | -0.1602*** | -0.1600*** |
|  | (0.0219) | (0.0221) | (0.0229) | (0.0230) |
| College and above | -0.1958*** | -0.1929*** | -0.2324*** | -0.2296*** |
|  | (0.0245) | (0.0248) | (0.0256) | (0.0259) |
| Kinh majority | 0.0768*** | 0.0769*** | 0.0408** | 0.0419** |
|  | (0.0181) | (0.0182) | (0.0187) | (0.0188) |
| Wealth index | -0.0248*** | -0.0251*** | -0.0444*** | -0.0445*** |
|  | (0.0079) | (0.0079) | (0.0082) | (0.0083) |
| Urban (urban=1; rural=0) | 0.0653*** | 0.0650*** | 0.0456*** | 0.0456*** |
|  | (0.0136) | (0.0137) | (0.0144) | (0.0144) |
| Provincial overall CPI | -0.0255* | -0.0394** | -0.0162 | -0.0237 |
|  | (0.0139) | (0.0155) | (0.0138) | (0.0151) |
| Log of population density of provinces | 0.0219 | 0.0334 | -0.0340 | -0.0323 |
|  | (0.0389) | (0.0412) | (0.0418) | (0.0435) |
| Log of per capita income of provinces | 0.0056 | 0.0016 | 0.0148** | 0.0095 |
|  | (0.0083) | (0.0079) | (0.0069) | (0.0079) |
| Province fixed-effects | Yes | Yes | Yes | Yes |
| Year fixed-effects | Yes | Yes | Yes | Yes |
| Observations | 93,662 | 86,834 | 93,662 | 86,834 |
| Pseudo R2 | 0.112 | n.a. | 0.119 | n.a. |
| Note: This table reports marginal effects of the explanatory variables from the probit and IV-probit models. The constants are included in the model, but not reported in this table. The marginal effects are computed using the “margin” command in Stata. For dummy variables, the marginal effect is the estimated discrete change in the probability of the dependent variable due to the change in the dummy variables from 0 to 1. For a continuous explanatory variable, it is the estimated partial derivative of the dependent variable with respect to the explanatory variable (evaluated at the mean value of all the other explanatory variables).  Robust standard errors in parentheses. Standard errors are corrected for sampling weights and cluster correlation within individuals and clusters.  *** p<0.01, ** p<0.05, * p<0.1.  Source: Estimation from GATS 2010 and 2015. | | | | |
